# Effect of SAM-Dependent Methyltransferases from *Halomonas* sp. YLGW01 on Phospholipid Fatty Acids Composition and Production of Polyhydroxalkanoates in *Escherichia coli*

**DOI:** 10.4014/jmb.2412.12065

**Published:** 2025-04-23

**Authors:** Tae-Rim Choi, Gaeun Lim, Yebin Han, Jong-Min Jeon, Jeonghee Yun, Jeong-Jun Yoon, Shashi Kant Bhatia, Yung-Hun Yang

**Affiliations:** 1 Department of Biological Engineering, Advanced Materials Program, College of Engineering, Konkuk University, Seoul 05029, Republic of Korea; 2 Green Circulation Research and Development Department, Korea Institute of Industrial Technology (KITECH), Cheonan 31056, Republic of Korea; 3 Department of Forest Products and Biotechnology, Kookmin University, Seoul 02707, Republic of Korea; 4 Institute for Ubiquitous Information Technology and Applications, Konkuk University, Seoul 05029, Republic of Korea

**Keywords:** *Halomonas*, phospholipid fatty acid, cyclopropane fatty acid, furan fatty acid, polyhydroxyalkanoate

## Abstract

The bacterial membrane changes in response to growth conditions such as salt concentration, temperature, and growth inhibitors. As membrane fatty acid related genes are important in controlling the membrane composition, we studied two genes *i.e.* cyclopropane fatty acid synthase (*cfa*) and *cis*-vaccinate 11-methyltransferase (*ufaM*) that designated to S-adenosylmethionine (SAM)-dependent methyltransferase derivatives from *Halomonas* sp. YLGW01. These two genes are related to a response to external growth conditions by controlling the fluidity of membrane changes. The role of these two genes (*cfa* and *ufaM*) was accessed by overexpression in *Escherichia coli* and results showed an evident increase in the levels of cyclic fatty acid and detection of furan fatty acid intermediate analyzed by Gas chromatography Mass spectrometer (GC-MS), respectively, resulting in changes of phospholipid fatty acids (PLFA) and membrane properties. Overexpression of these genes in engineered polyhydroxyalkanoate (PHA) producing *E. coli* strain promoted the bacterial growth (1.6-fold) and PHA accumulation (2-fold). Overall, this study showed two membrane fatty acid synthases, *ufaM* and *cfa* able to change membrane fatty acid composition and have the potential to improve cell's robustness and PHA production capability.

## Introduction

The marine environments had complex and harsh conditions, and marine bacteria adapted themselves to grow athigh salt concentrations, low temperatures (below 4°C), and nutrient depletion [1-3]. The halotolerant and halophilic bacteria and the mechanisms by which these organisms adapted to osmolarity have been extensively studied, and osmotic-related survival mechanisms such as membrane composition control is the main mechanism [4-6].

*Halomonas* is a popular bacterium that exists in saline environments around the world and has a tolerance to NaCl ranging from 0.1 to 32.5% [7]. To endure saline conditions and other stress conditions, they could control the osmotic balance by increasing the concentration of an ectoine or accumulate a lot of PHA granules inside the cell [8-10]. Therefore, *Halomonas* is a promising strain for industrial use as well as for investigating the mechanisms to deal with osmotic stress [11].

Fatty acid metabolism is altered in response to gene expression and several studies have attempted to figure out specific targets that are related to the membrane fatty acids modification [12, 13]. Some interesting genes such as cyclopropane fatty acid synthase (*cfa*) and Δ-9-fatty acid desaturase (*desA*) codes enzymes involved in biosynthesis of unsaturated fatty acids which play an important role in membrane properties [14]. Furthermore, some marine bacteria have furan fatty acids as a unique fatty acid that can scavenge hydroxy radicals a causing agent of stress [15-17].

S-adenosylmethionine (SAM)-dependent methyltransferase is a one group of SAM-dependent enzymes that catalyze various types of reactions [18]. One SAM-dependent methyltransferase is associated with cyclopropane fatty acid synthesis, such as cyclic fatty acid synthases (CFAs) [18]. For another fatty acid synthesis, other type of enzyme, such as *UfaM*, which incorporates with furan fatty acid synthesis [19]. The overexpression of this SAM-dependent methyltransferase has been shown to enhance microbial growth, but at the same time, a study has observed a decrease in solvent production, highlighting the need to explore its potential applicability for the production of other metabolites [20].

Polyhydroxyalkanoates (PHAs) are biodegradable polymers accumulated in intracellular granules by many bacteria as a reserve material [21]. These polymers have been extensively studied due to their thermoplastic properties which make them attractive candidates for the replacement of traditional plastics [22]. The basic conditions for microorganisms to accumulate PHA are the accumulation of carbon in the body to survive in stress conditions such as nitrogen limitation, carbon surplus, oxygen limitation, and cold shock [23-25]. In addition to simply accumulating carbon, some studies show that PHAs increase resistance to external stress [26, 27], just like chaperones [28]. As a response to external stress, bacterial membrane composition changes and PHA accumulation were compared to confirm their relationship on growth [29]. To enhance microbial robustness against various stresses present in PHA production environments and achieve high microbial growth, a strategic approach involving modifications to membrane fatty acid composition was studied [30, 31].

In this study, two SAM-dependent methyltransferase genes were characterized by marine bacteria *Halomonas* sp. YLGW01 isolated from marine soil, which was found to show changes in membrane phospholipid fatty acid (PLFA) content by synthesizing cyclopropane fatty acid and an intermediate of furan fatty acid. The role of two genes was determined as *cis*-vaccinate 11-methyltransferase (*ufaM*) and cyclopropane fatty acid synthase (*cfa*) using PLFA analysis and phylogenetic comparison based on multiple sequence alignment. Two genes were applied for observing various changes in *E. coli*. The results of these experiments are expected to distinguish two similar fatty acid synthases and to provide approaches to increase bacterial growth and PHA accumulation as a synergetic effect to deal with harsh growth conditions.

## Material and Methods

### Chemicals

All chemicals used in the present study were of analytical grade or higher. Fatty acids such as palmitic acid and stearic acid were purchased from Sigma-Aldrich (USA). Other chemicals used in the growth media were also purchased from Sigma-Aldrich or BD Difco (USA).

### SAM-Dependent Methyltransferase Gene Screening and Phylogenetic Analysis

The genome data of bacterial strain *Halomonas* sp. YLGW01 was already deposited on NCBI as accession number CP062005.1 and taxonomy ID 2773308 [32]. Two SAM-dependent methyltransferase sequences were listed in Table S1. Sequences of various cyclopropane fatty acid synthases were compared with the genome of *Halomonas* sp. YLGW01 using a basic local alignment search tool (BLAST). Further comparison of detected sequences was conducted by using MEGA X program to make a phylogenetic tree for protein sequences.

### Strains and Cultural Conditions

*Halomonas* sp. YLGW01 was cultured in marine broth 2216 (MB) at 30°C and 200 rpm for pre-culture to use further in experiments [10]. *Escherichia coli* strains were cultured using LB media, supplemented with 50 μg/ml of kanamycin, 100 μg/ml of spectinomycin, and 0.1 mM of IPTG if needed, at 30°C. The two SAM-dependent methyltransferase genes of *Halomonas* sp. YLGW01 (*ufaM* and *cfa*) were cloned into pET24ma and transformed into *E. coli* for further experiments. Table 1 lists the strains used for gene overexpression and the primers used for cloning the two genes.

### PHA Producing Condition

Various SAM-dependent methyltransferases derived from different species were cloned to pET24ma and transformed to *E. coli* with pLW487 for observing changes in bacterial growth and PHA accumulation [33]. The engineered *E. coli* strains for PHA production experiments were pre-cultured in LB medium at 30°C supplemented with 50 μg/ml of kanamycin, and 100 μg/ml of spectinomycin if needed. The main culture for the PHA production was M9 minimal media, 2% of glucose, and 0.1% of yeast extract supplemented with 50 μg/ml of kanamycin, 100 μg/ml of spectinomycin, and 0.1 mM IPTG as needed at 30°C. A culture time and sampling points were varied 24 h to 72 h for the experiment purpose.

### PLFA Analysis with GC-MS

The PLFA composition of *Halomonas* sp. YLGW01 and engineered *E. coli* was evaluated using PLFA analysis method as already reported [34, 35]. The strains were cultured in marine broth or LB medium at 30°C and 200 rpm for 72 h. The culture broth was centrifuged (3500 *g* at 4°C, up to 30 min) and washed twice with deionized water. The washed cells were transferred to a glass vial for lyophilization. Lipid extraction and further experiments were conducted based on the Bligh and Dyer method and the MIDI protocol [36, 37]. Briefly, 5 ml of the lyophilized culture of microbes was used to which 2 ml of methanol and chloroform were added, and mixture was vortexed at 25°C for 2 h for lipid extraction. After incubation 2 ml distilled water was added to the mixture followed by vortexing and centrifugation at 1500 *g* for 5 min. 2 ml of the liquid phase was transferred to glass vials and the sample was evaporated with N_2_-gas and re-treated with 1 ml of chloroform for further steps. Samples were prepared by using mild alkaline methanolysis of phospholipids, 0.5 ml methanol, 0.5 ml toluene, and 1 ml 0.3 M methanolic-KOH were added to the samples followed by incubation at 37°C for 15 min. The organic phase was then extracted and transferred into clean borosilicate glass tubes containing 1 mg of Na_2_SO_4_. The resulting samples were analyzed by GC-MS (Perkin Elmer) equipped with a fused silica capillary column (Elite-5 ms, 30 m, 0.25 mm, i.d. 0.25-μm film) and subjected to a linear temperature gradient for fatty acids (120 °C held for 5 min, increased at 6°C/min to 200°C, increased at 2°C/min to 220°C, and then increased at 10°C/min to 300°C). The injector port temperature was set at 210°C. Mass spectra were obtained by electron impact ionization at 70 eV, and scan spectra were obtained within the range of 45-400 m/z. Selected ion monitoring was used for the detection and fragmentation analysis of the major products [38].

### Gas Chromatography for PHA Quantification

PHA was quantified and characterized using Gas chromatography – Flame Ionizer Detector (GC-FID; Youngin Chromass, Republic of Korea) according to a previously described method with small modification [39]. In brief, the culture broth was centrifuged and washed twice with water. The washed cells were transferred into a glass vial for lyophilization, and the dry cell weight was measured. Equal volumes of chloroform and 15% (v/v) H_2_SO_4_/85%methanol solution (2 ml total volume) were added to the glass vial, and methanolysis was performed for 2 h at 100°C, followed by cooling to room temperatures. A 1-ml aliquot of deionized water was added to the methyl ester solution, which was vortexed for 5 s. The chloroform layer was transferred into a microtube containing crystalline anhydrous Na_2_SO_4_ to remove the residual water. Filtered 1 μl aliquots were injected into a gas chromatograph in split mode (1/10) (Young-lin 6500, Republic of Korea), equipped with a fused silica capillary column (Agilent HP-FFAP, 30 m × 0.32 mm, i.d. 0.25 μm film) and a flame ionization detector (FID). The inlet temperature was 210°C, and helium was supplied as the carrier gas at a rate of 3 ml/min. The oven temperature was controlled following a gradient program of 0-5 min at 80°C and 12-17 min at 220°C. The FID temperature was maintained at 230°C throughout the experiments.

### Gel Permeation Chromatography

Gel permeation chromatography was used to measure the number average molecular weight (Mn), the weight-average molecular weight (Mw), and the polydispersity index (PDI). The sample preparation and operation of the GPC were slightly changed based on the previous report [40]. Briefly, The gel permeation chromatography (Youngin Chromass) an HPLC system consist of a loop injector (Rheodyne 7725i), an isocratic pump system with dual heads (YL9112), a column oven part (YL9131) with three columns (K-G 4A, guard column; K-804 8.0 × I.D. × 300 mm; K-805, 8.0 I.D. × 300 mm; respectively, Shodex), and a refractive index detector (YL9170). As a mobile phase, chloroform with 1 ml/min flow rate was used at 40°C. Injection volume was 20 μl of prepared sample. For the calculation of the molecular weight, polystyrene standards range from 5,000 to 2,000,000 Da were used to set calibration curve.

### High Performance Liquid Chromatography

Residual glucose was analyzed by high performance liquid chromatography (HPLC; PerkinElmer, USA) system equipped with a refractive index detector (RID; PerkinElmer). Separation of the injected sample was performed on an Aminex HPX-87H column (300×7.8 mm i.d.; Bio-Rad, USA). The mobile phase in which the flow rate was maintained at 0.6 ml/min was 0.008N H_2_SO_4_; oven temperature was maintained at 60°C throughout the operation [41].

## Results and Discussion

### Finding of SAM-Dependent Methyltransferase Genes from *Halomonas* sp. YLGW01

**Fig. 1:**
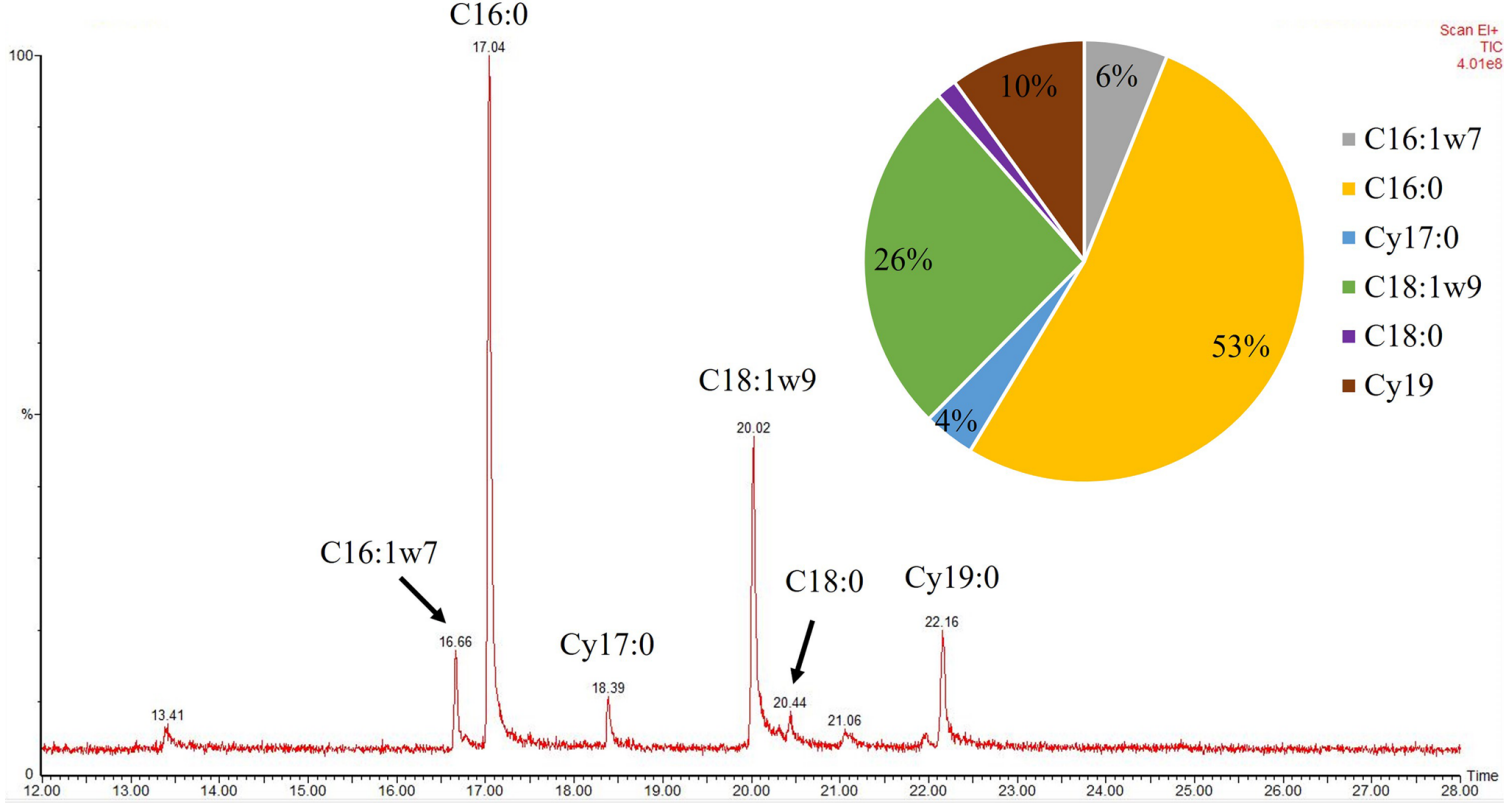
Phospholipid fatty acid pattern of the marine bacteria *Halomonas* sp. YLGW01.

A halophilic marine bacterium *Halomonas* sp. YLGW01 was studied as hyper PHA producing strain and whole genome sequencing was done in previous report for a better understanding of the strain [32]. To find out the robustness of novel strain as halophilic characteristic of the bacterium, phospholipid fatty acid composition as a bacterial membrane also observed by GC-MS and compared with other gram-negative bacteria. Due to changes in growth condition of bacteria such as temperature and salt concentration lead reactions on bacterial membrane, especially with phospholipid fatty acid composition change [42, 43]. In optimal growth condition, *Halomonas* sp. YLGW01 had portion that 4% of 9,10-methylenehexadecanoic acid (Cy17:0) and 10% of *cis*-9,10-methyleneoctadecanoic acid (Cy19:0) found in bacterial cell membrane (Fig. 1). Based on information of *Pseudomonas* sp. B14-6 and *Halomonas socia*, possible membrane related genes were searched in the *Halomonas* sp. YLGW01 genome. By processing a whole genome-based BLAST with membrane associated genes such as *cfa* and *desA*, two highly matched genes in *Halomonas* sp. YLGW01 were found (YLGW01_00254 and YLGW01_01171). Two genes were categorized as SAM-dependent methyltransferase that played an important role in cyclopropane fatty acid biosynthesis and the possibility for the presence of furan fatty acid [44].

### Identification of Two Membrane Fatty Acid-Modulating Enzymes in the *Halomonas* sp. YLGW01

**Fig. 2:**
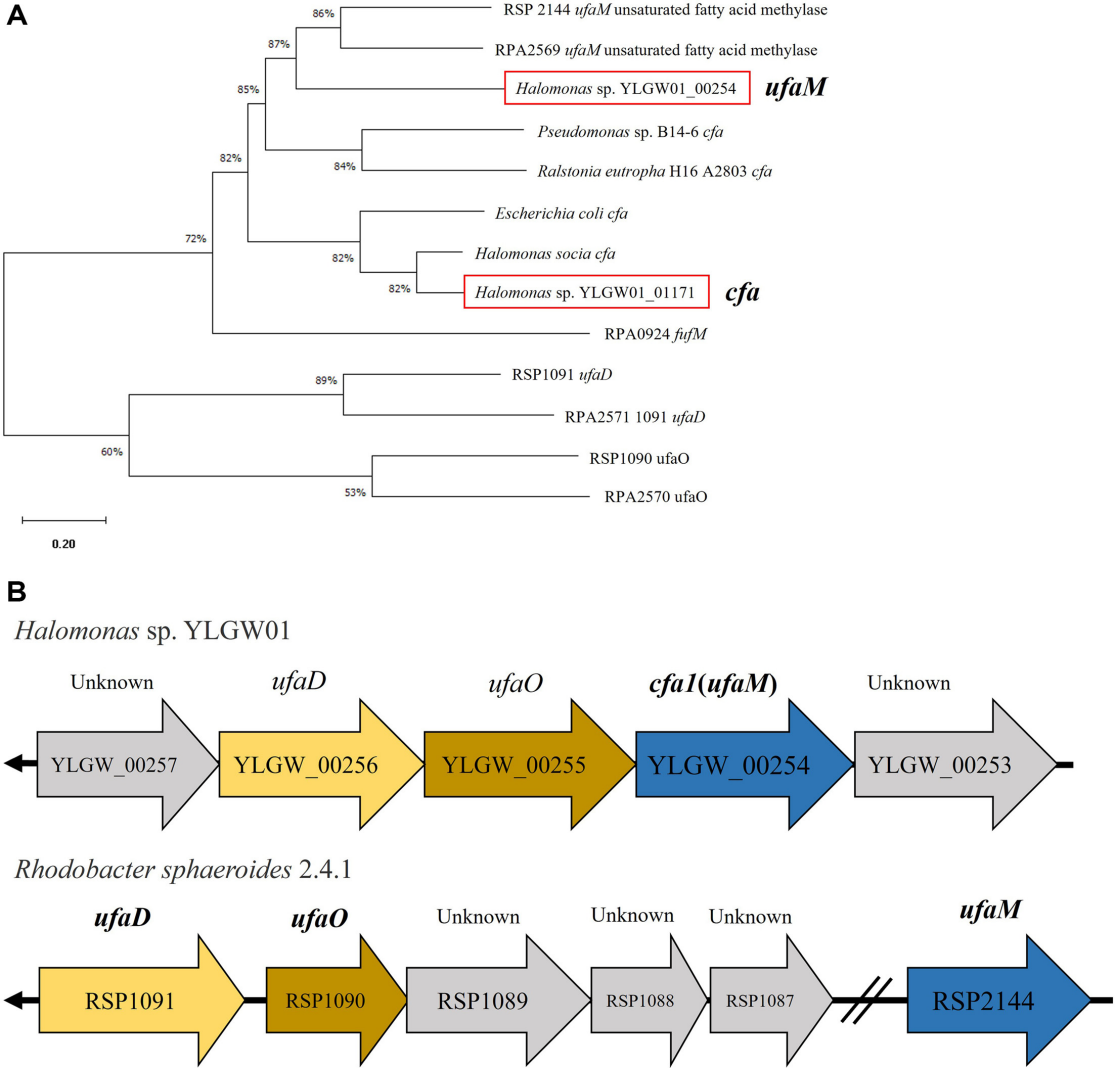
Identification of new *cfas* from *Halomonas* sp. YLGW01. (**A**) Phylogenetic analysis of two SAM-dependent methyltransferases to other membrane related fatty acid synthases from various species. (**B**) A comparison of furan fatty acid synthesis genes to *R. sphaeroides* for *ufaM*.

As further research about membrane fatty acid related genes, especially SAM-dependent methyltransferase derivatives, we compared two SAM-dependent methyltransferase gene sequences of *Halomonas* sp. YLGW01 to the sequences of other bacteria including *Pseudmonas*, *E. coli*, and *Halomonas*. One gene was discovered that had a high match to *Halomonas socia* *cfa* gene. However, the other gene had a much lower match with *cfa* gene sequences. Therefore, we assumed that one gene works as cyclopropane fatty acid as already studied and the other works as *ufaM* that related to the furan fatty acid biosynthesis [19]. Two target gene sequences were compared to cyclic fatty acid synthesis and furan fatty acid synthesis genes. As a result, YLGW01_00254 was found to be *cis*-vaccenate 11-methyltransferase (*ufaM*) and YLGW01_01171 was the previously studied cyclopropane fatty acid synthase (*cfa*) (Fig. 2A). Some reports revealed furan fatty acid synthesis process and related genes including *ufaM*, here the furan fatty acid synthesizing genes in *Halomonas* sp. YLGW01 was also observed (Fig. 2B) [19]. Although *ufaM* of *Rhodobacter sphaeroides* 2.4.1 was far from other genes *ufaD* and *ufaO*, three genes of *Halomonas* sp. YLGW01 were consecutive.

### Study of Effect on Membrane Composition by Overexpression of *ufaM* and *cfa* in *E. coli*

**Fig. 3:**
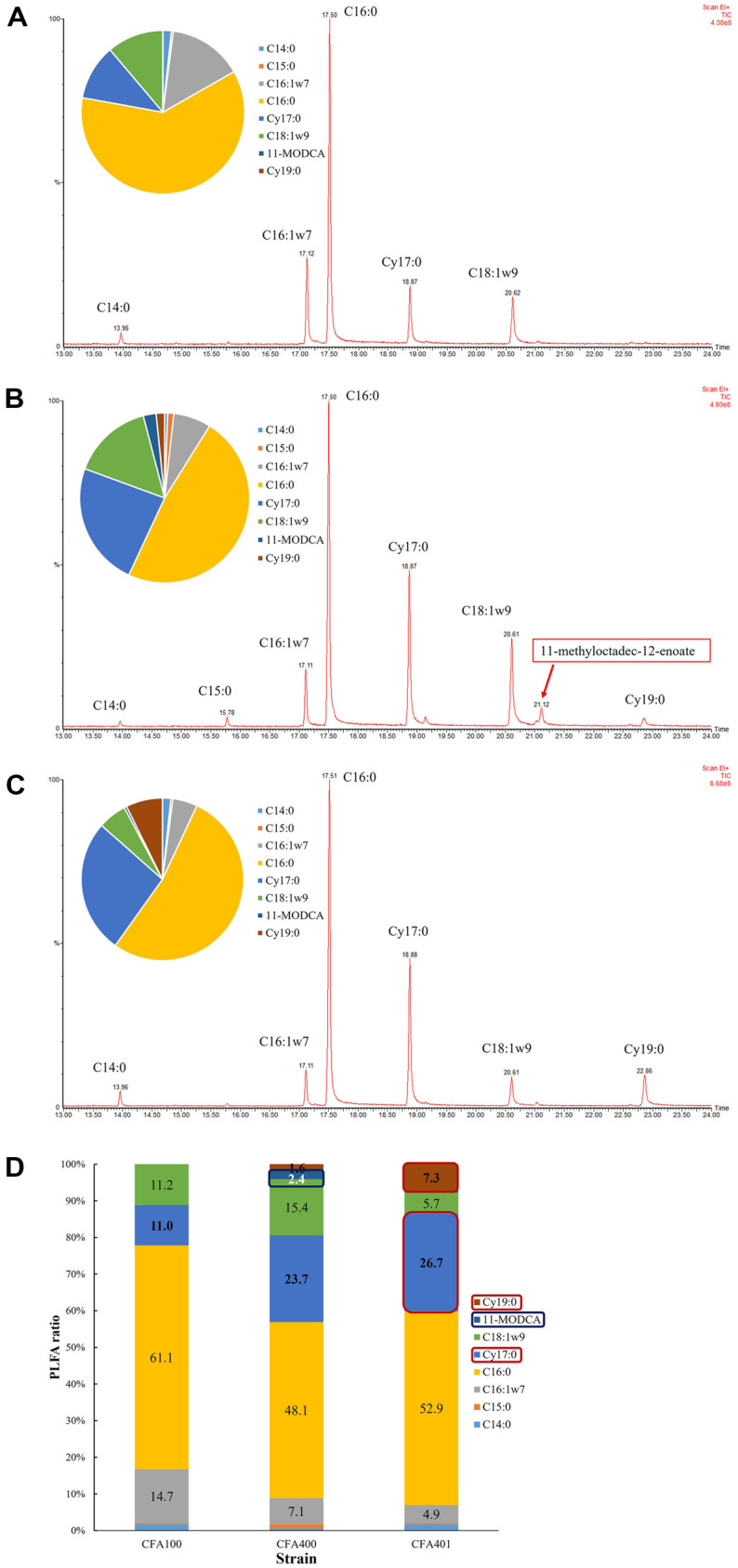
PLFA analysis and membrane characterization of *cfa*-overexpressed *E. coli*. PLFA patterns of engineered strains that are cultured. (**A**) control strain harboring pET24ma vector, (**B**) pET24ma::*ufaM* harboring KSYH(DE3) strain and (**C**) pET24ma::*cfa* harboring KSYH(DE3). (**D**) PLFA pattern comparison of three strains with bar graph. Blue arrow indicating 11-MOCDA synthesized by *ufaM* overexpression and red arrows indicating cyclic form fatty acids that were associated with *cfa* overexpression.

**Fig. 4:**
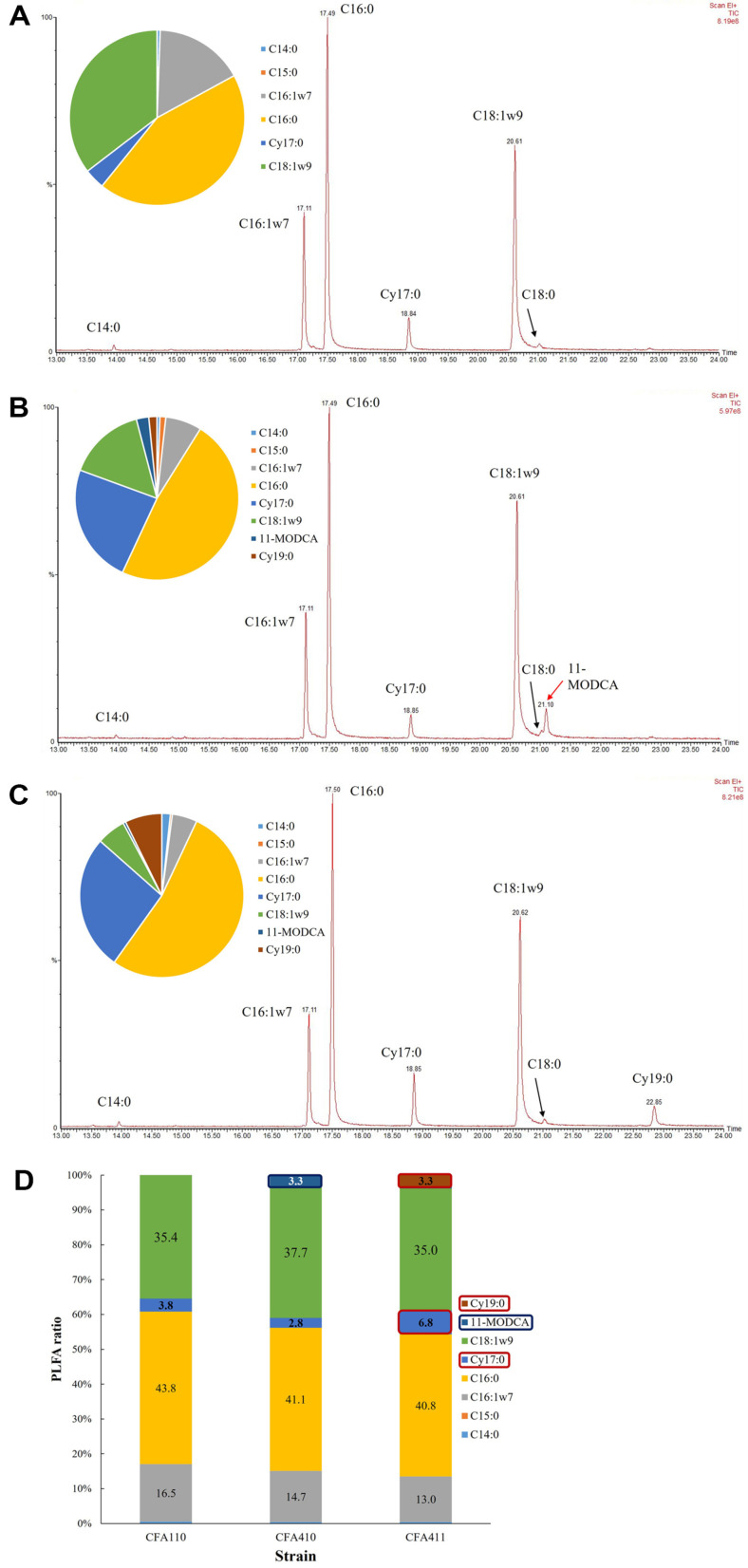
PLFA analysis and membrane characterization of *cfa*-overexpressed PHA producing *E. coli*. (a) PLFA patterns of engineered strains that cultured. (**A**) control strain harboring pET24ma vector and pLW487. (**B**) Harboring pET24ma::*ufaM* and pLW487 in BL21(DE3) strain and (**C**) pET24ma::*cfa* and pLW487 harboring BL21(DE3). (**D**) PLFA pattern comparison of three strains with bar graph. Blue arrow indicating 11-MOCDA synthesized by *ufaM* overexpression and red arrow indicating cyclic form fatty acids that were associated with *cfa* overexpression.

PLFA composition and its modification were important data for cell viability changes and related genes for each fatty acid synthesis [45]. *UfaM* and *cfa* from *Halomonas* sp. YLGW01 introduced in *E. coli* to figure out which fatty acids are directly affected by *ufaM* and *cfa*, respectively. When compared to the control strain PLFA pattern (Fig. 3A), PLFA of *ufaM* overexpressing strain showed a new peak assigned to 11-methyloctadec-12-enoate (11-MODCA)(Fig. 3B). Similarly, *cfa* overexpressing strain had much higher cyclic form fatty acids such as *cis*-9,10-methylenehexadecanoic acid (Cy17:0) from 11.0% to 26.7% and methyleneoctadecanoic acid (Cy19:0) from 0%to 7.3% (Fig. 3C). When comparing all strains, the 11-MODCA peak was detected only at *ufaM* overexpression strain and cyclic fatty acids were detected in both *ufaM* and *cfa* overexpression strain (Fig. 3D). The changes were similar in PHA producing strain with *ufaM* or *cfa*. When compared to control strain (Fig. 4A), *ufaM* strain had 11-MODCA peak (Fig. 4B) and *cfa* strain had more cyclic fatty acids (Fig. 4C). Although other major fatty acids were almost similar, specific fatty acids that related to *ufaM* and *cfa* were different (Fig. 4D). The results demonstrated that overexpression of the two SAM-dependent methyltransferases derived from *Halomonas* sp. YLGW01 could accelerate changes in cell membrane fatty acid composition by making cyclic form of phospholipid fatty acid which was normally showed in the stationary phase or the stress condition [46]. This altered composition was expected to enhance cell robustness, thereby promoting increased microbial growth [47, 48].

### Effect of *ufaM* and *cfa* Overexpression for PHA Production in *E. coli*

**Fig. 5:**
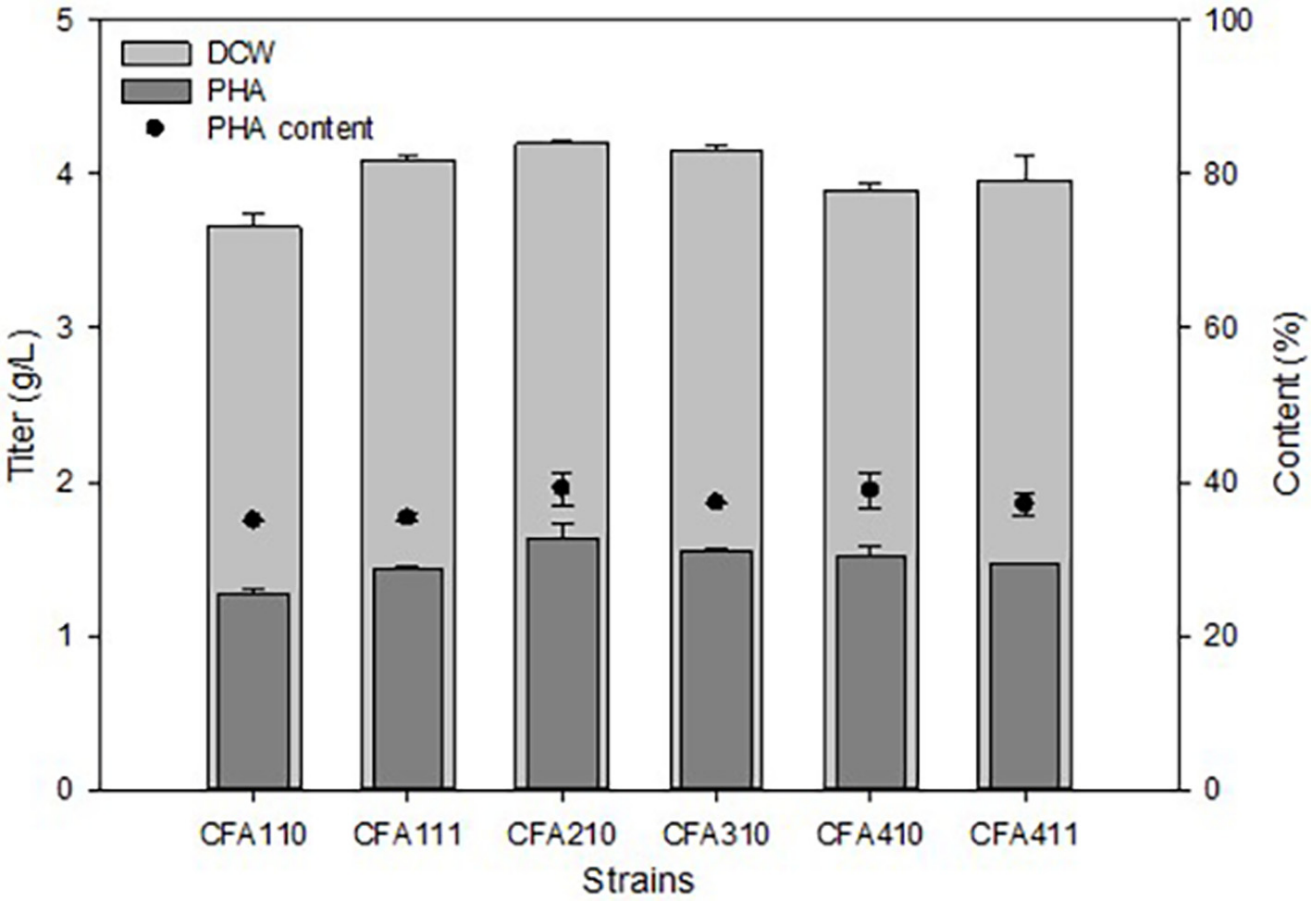
Changes in cell growth and PHA production by overexpression of *cfa* in *E. coli* A comparison of PHA production using different SAM-dependent methyltransferases derived from various species. As strain number, 1: CFA110; 2: CFA111; 3: CFA210; 4: CFA310; 5: CFA410; 6: CFA411.

**Fig. 6:**
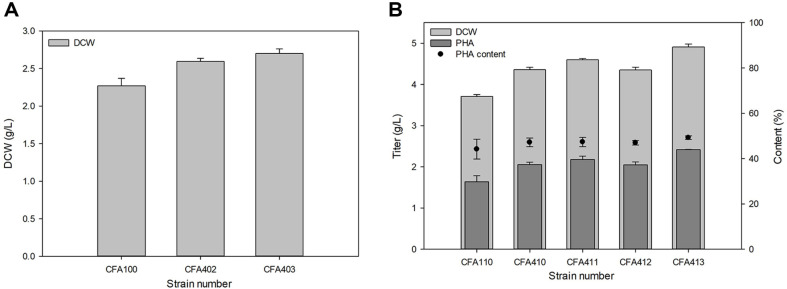
Changes in PHA production by single and double overexpression of *cfa* in *E. coli*. Comparison of engineered *E. coli*. (**A**) Simple growth change as numbered, 1: CFA100; 2: CFA402; 3: CFA403. (**B**) PHA producing condition for strain number 1: CFA110; 2: CFA410; 3: CFA411; 4: CFA412; 5: CFA413.

Our previous study suggested that overexpression of *cfa* in PHA producing engineered *E. coli* could increase cell growth and PHA production [33]. However, the amount of increment could be varied for the bacterial strain. Therefore, we compared various *cfa* genes for improving PHA production. *ufaM* and *cfa* from *Halomonas* sp. YLGW01 were better than the control but not than other *cfas* such as *E. coli*, *Pseudomonas* sp. B14-6 and *Halomonas socia* (Fig. 5). To confirm their synergetic effect in overexpression, designed experiments that overexpressed two genes in one *E. coli*. One approach was cloning two genes in one vector and the other was one gene per one vector. Without the PHA accumulation, all double overexpressed strains were better in growth (Fig. 6A). However, in the case of PHA production *cfa*-*ufaM* cloned vector harboring strain had improved growth and PHA content (Fig. 6B). Through the overexpression of the two genes, the proportion of cyclopropane fatty acids and furan fatty acid intermediates was maintained at higher levels compared to the control. This higher proportion contributed to relatively increased microbial robustness, preventing cell death and resulting in an elevated dry cell weight, which corresponded to a proportional increase in PHA accumulation.

### Time Dependent Monitoring of PHA Production with *cfa* and *ufaM* Overexpression

**Fig. 7:**
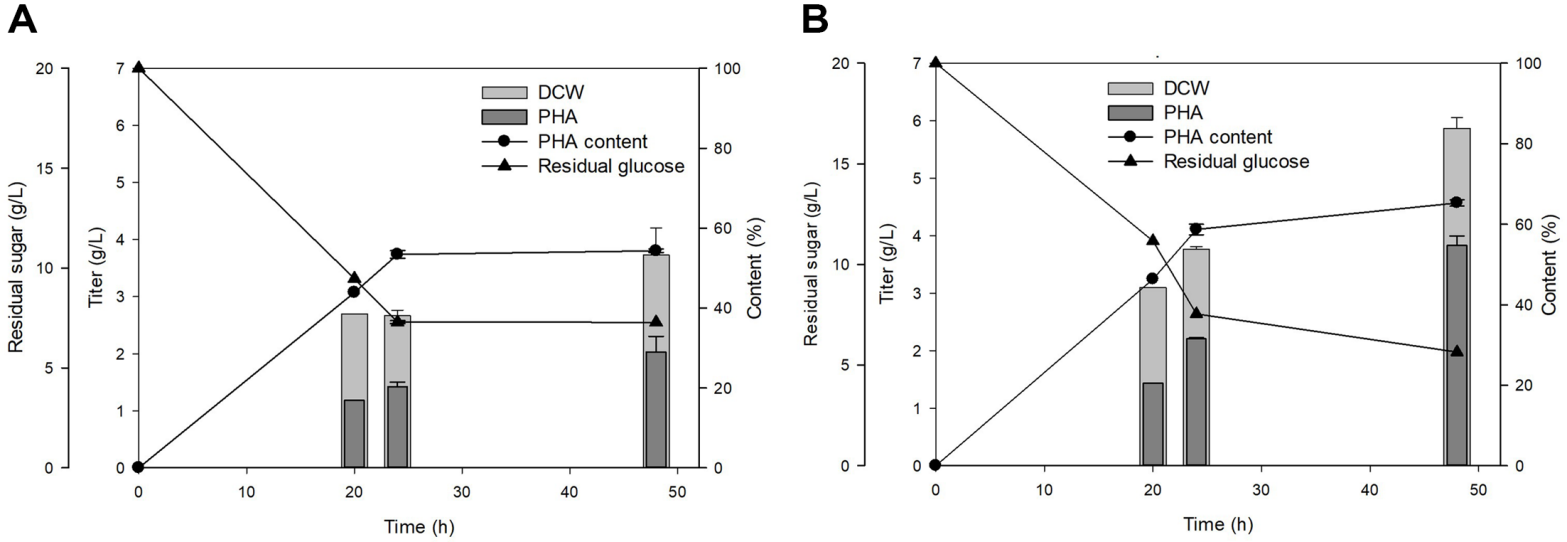
Time-dependent results in flask-scale PHA production of *E. coli* with overexpression of both *cfas* in single vector and double vector system. (**A**) control strain (CFA110) growth pattern in cultivation for 48 h and (**B**) CFA413.

Overexpression of *ufaM* and *cfa* were confirmed to increase cell growth and PHA accumulation. The culture volume increased to the flask level to confirm the changes and produced PHA extraction for further analysis. The control strain CFA110 was compared to engineered strain CFA413 (*cfa*-*ufaM*). The control strain had 3.7 g/l DCW and 52% of PHA content (Fig. 7A). CFA413 strain had a higher 6 g/l of DCW and 65% of PHA content (Fig. 7B). In conclusion, CFA413 had the best as PHA producing strain with SAM-dependent methyltransferase overexpression. In further experiments with extracted PHA, GPC analysis was conducted to observe the differences in molecular weight in gene overexpression (Table 2). Both strains had a decrement in the molecular weight of PHA with culture progress. The molecular weight was lower in CFA413 although it had better biomass titter and PHA content. This phenomenon was similar to the work of phasin protein overexpression that made smaller PHA granules and higher PHA content, considering about PHA content of the overexpression strain; granules inside the cells became smaller but occupied more space, as observed by TEM [49].

## Conclusion

To find out the robustness of halophilic bacteria, the genome of the marine stain *Halomonas* sp. YLGW01 was analyzed to search for membrane fatty acid synthesis associated genes, especially cyclopropane fatty acid. By alignment of various *cfa* sequences from *Pseudomonas*, *Halomonas* and *Escherichia*, two SAM-dependent methyltransferase genes were identified using phylogenetic analysis and PLFA analysis. Both *ufaM* and *cfa* had direct effects on membrane fatty acids composition shown by the presence of 11-methyloctadec-12-enoate (11-MODCA) and cyclic form fatty acid (Cy17:0 and Cy19:0), respectively. Their overexpression in engineered *E. coli* slightly increased cell growth and PHA accumulation and results demonstrate that changes in membrane composition were highly related to resistance to environmental stress. Further, the production of rare furan fatty acids using *ufaM* and related genes could have potential in fatty acid production and application.

## Supplemental Materials

Supplementary data for this paper are available on-line only at http://jmb.or.kr.



## Figures and Tables

**Table 1:** Strains and plasmids.

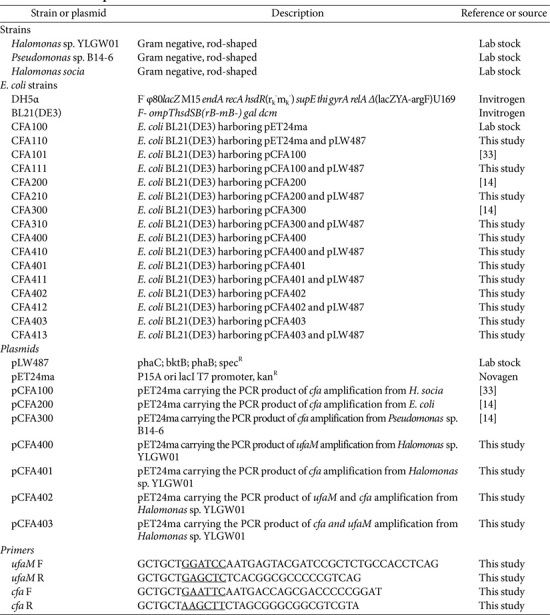

**Table 2:** GPC results for PHA molecular weight extracted from engineered *E. coli*.

		Max RT	Mn	Mw	PDI
CFA110	24	12.24	1471760	1747263	1.1872
	48	12.31	1278703	1579719	1.2354
CFA413	24	12.28	1234598	1550564	1.2559
	48	12.4	1019054	1350166	1.3249
