# Supplementary material for: Effect of SAM-Dependent Methyltransferases from Halomonas sp. YLGW01 on Phospholipid Fatty Acids Composition and Production of Polyhydroxalkanoates in Escherichia coli
Source: J Microbiol Biotechnol. 2025 Apr 23;35:e2412065. doi: 10.4014/jmb.2412.12065 (PMC12089945; doi:10.4014/jmb.2412.12065)
Supplement: Supplementary file 1 [file jmb-35-e2412065-supple.pdf]

J. Microbiol. Biotechnol. 2025. 35: 00 e2412065

https://doi.org/10.4014/jmb.2412.12065

Supplementary Table

Effect of SAM-dependent methyltransferases from *Halomonas* sp. YLGW01 on phospholipid fatty acids composition and production of polyhydroxalkanoates in *Escherichia coli*

Tae-Rim Choi[1,2], Gaeun Lim[1], Yebin Han[1], Jong-Min Jeon[2], Jeonghee Yun[3], Jeong-Jun Yoon[2], Shashi Kant Bhatia[1,4*], and Yung-Hun Yang[1,4*]

[1]Department of Biological Engineering, Advanced Materials Program, College of Engineering, Konkuk University, Seoul 05029, Republic of Korea

[2]Green Circulation Research and Development Department, Korea Institute of Industrial Technology (KITECH),
Cheonan 31056, Republic of Korea

[3]Department of Forest Products and Biotechnology, Kookmin University, Seoul 02707, Republic of Korea

[4]Institute for Ubiquitous Information Technology and Applications, Konkuk University, Seoul 05029, Republic of Korea

*Corresponding authors:

Yung-Hun Yang / E-mail: seokor@konkuk.ac.kr

hashi Kant Bhatia / E-mail: shashibiotechhpu@gmail.com

Table S1. Sequneces of two SAM-dependent methyltransferases of *Halomonas* sp. YLGW01

Gene	Sequence
YLGW01_ 00254 (*ufaM*)	GTGAGTACGATCCGCTCTGCCACCTCAGCGCCGCAGCCTGATACCGGCGAT<br>CGCCTGACCCGACTGCTCAAGCCCCGCCTGCTCGCCCAGCTGGCCGATCTC<br>GAGGGCGGCCAGATCACCCTGATCGAGGGCGATCAGCGCCATGAACTGGG<br>CCGCGGCGGGCCGCTCTCGGTCACAGTCGTGGTCAATCGCCCCCAGGCCT<br>GGCGCCGGCTGGCGCTGGGCGGCACGGTGGGCGCCGCCGAGGCCTACATG<br>GATGGCGATTGGGAGGCCGATGACCTGGTCGCTCTGACCCGGCTGTTCGCC<br>GCCAATCTGGAACGGGTCAACGGCGAGGTCGAGAACGGCAGCGCGCGCT<br>TCGGTCGCTGGCTGCTGACCGCTGCCTACGCCCTGCAGCGCAACACTCAG<br>CGCGGCGCCCGGCGCAACATCTCGGCCCACTATGATCTTGGCAACGAGCTG<br>TTCGCGACCTTCCTCGACACCGAGCACTGGATGTACTCCAGCGCCATCTTC<br>CCGCACCCGGAGGCGAGCCTCGAGGAGGCCTCGACCCATAAGCTCGATGT<br>GATGCTCGACCGGCTCGACGTGGGCCCCGAGCACCACCTGCTGGAGATCG<br>GCACCGGCTGGGGCGGGCTCGCCCTTCACGCCGCCAAGAGCCGCGGCTGC<br>CGGGTCACCACCACCACCATCTCCGACGAGCAATATGCCCACACCGCGGC<br>ACGCATCGAGGAAGAGGGGCTCGGCGAGCGGATCACCCTGCTCAAGCAG<br>GACTACCGGGATCTCGAGGGCCGCTATGATCGGGTGATCTCGGTGGAGATG<br>ATCGAGGCGGTGGGGCATCAGTACCTCAACACCTATCTCGCCACCCTGGAC<br>CGGCTGCTCACCGACGACGGCCTGGTCATGCTGCAGGCGATCACCATCCGC<br>GACCAGCGCTTCGAGGCCGCCAAGCGCGAGATGGACTTCATCAAGCGCTA<br>CATCTTCCCCGGCGGTTTCCTGCCCTCGCACCGCGCCATCCTCGACGGCAT<br>CACCCGCCATACCTCGCTGAACGTGCTGTCGCTTGACGAGATCGGGCTGCA<br>CTATGCGCGCACCTTGCGAGAGTGGCGTCATCGCTTCGAAGCTCGCCTGGA<br>GCGGGTCCGCAAGCTCGGCTACGACGAACGCTTCATTCGCATGTGGCGCTA<br>TTACCTGTGCTACTGCGAGGGCGGTTTCCTGGAGCGCAGCATCGGCACCTG<br>CCACCTGCTGATGGCCAAGCCCGGTGCTCGCCGCGATGCCCTGACGGGGG<br>CGCCGTGA
YLGW01_ 01171 (*cfa*)	ATGACCAGCGACCCCCGGATCGGTGCCATCGCCTTACCCGACACCCGTGCC<br>CGACGCATCGTCGAGCGGCTACTGGAGGGCTCGGGCGTGGCCCTGAACGG<br>CGGAGCGCCCTGGGACATGCAGGTCCTGCATCCCGACCTCTTCTCCAGGCT<br>GTTGCATCAAGGCACTCTAGGACTCGGGGAAGCCTACATGGAGGGCTGGT<br>GGCAGTGCGAGCGGATCGACGAGATGATTCATCGCATGTTGCGCCATGGCC<br>TTGGCGAGCGCGCCCACACGCCCTCGGAGAGGATGCTGTACCGCCTGCAG<br>ACCGGCCTCTTCAACCTGCAGAGCAAGGCCCGCGCCTACATCGTCGGCGA<br>GGCGCATTACGATCTGGGTAACGATCTCTTCGAGCGCATGCTCGACCCGAC<br>TCTCTGCTATTCCTGCGGTTACTGGAAGGAGGCCAACAGCCTGCACGAGGC<br>GCAGCTGGCCAAGCTGGACCTGGCCGCTCGCAAGCTGGGGCTTGCCCCGG<br>GCATGCACGTACTGGACATCGGCTGCGGCTGGGGCAGTTTCGCCGAACATG<br>CGGTGCGCCACTACGGTGTCGAGGTCACCGGCATCACCATTTCCCGGGAA<br>CAGGCCGAGCTGGCCCGCAGACGCTGCCAGGATTTGCCCGTCACCATCCT<br>GCTTCAGGACTACCGGGAGCTCGAAGGCCACTATGACCGTATCGTCTCGAT<br>CGGCATGTTCGAACACGTTGGCCATCGCAACTACCGCACCTACTTCGACAC<br>CGTGTCGCACCTGCTGGTCCCCGACGGGCTCTTCCTCTTGCACACCATCGG<br>CTCCAACAATTCGGGCATCAGCGCCGACCCCTGGATCCACAAGTACATCTT<br>CCCCAACGGGGTGCTGCCCTCGGCCATGCACCTCGCCCGCGCCAGCGAAC<br>CCTATCTGCTGATGGAGGACTGGCAGAACTTCGGCGCCGACTACGATCACA<br>CCCTGATGGCCTGGCTTCAGAACTTCGATGCCCGATGGCCGGAAATCGCCG<br>AACGCTACAACGAGACCACGCGTCGCATGTTCCGCTACTACCTCTCCGCCT<br>GCGCCGGCGCCTTCCGGGCGCGCGACCTGCAGCTCTGGCAGGTGGTCTTT<br>TCCCGGGGGCGCGAGGGACGCTACGACGCCGCCCGCTAG
